# Role of Vitamin D and Vitamin D Polymorphisms in COVID-19 Risk and Severity in Children: A Systematic Review

**DOI:** 10.7759/cureus.61326

**Published:** 2024-05-29

**Authors:** Victoria Giatraki, Emmanouil Galanakis, Chrysoula Perdikogianni

**Affiliations:** 1 Department of Paediatrics & Laboratory of Child Health, Medical School University of Crete, Heraklion, GRC

**Keywords:** immunoregulation, vitamin d, sars-cov-2, mis-c, covid-19, children

## Abstract

The role of vitamin D in the susceptibility to coronavirus disease 2019 (COVID‐19) disease has been investigated since the beginning of the pandemic, but there is still scarce data on children. We investigated the impact of vitamin D status and the related genetic variants on COVID-19 vulnerability and severity of the disease in children. A systematic review was performed in accordance with the Preferred Reporting Items for Systematic Reviews and Meta-Analyses (PRISMA) guidelines, to identify reports on vitamin D status and genetic polymorphisms, their association with the susceptibility of children to COVID-19 and multisystem inflammatory syndrome in children (MIS-C), and the effect of supplementation on the clinical course. Of an initial total of 279 articles, 26 studies, published between September 2020 and May 2023, were finally included in this review according to inclusion criteria. Quantitative data provided by 11 studies revealed that 43.05% of pediatric COVID-19 patients had low vitamin D levels. Mean serum 25(OH)D levels were observed to be significantly low in COVID-19 cases, with an estimated pooled mean value of 17 ng/mL, as provided by 16 studies. Vitamin D deficiency and the vitamin D receptor (VDR) FokI polymorphism may suggest independent risk factors for susceptibility to COVID-19 in the pediatric population. The 25(OH)D level may constitute a significant biomarker associated with the COVID-19 severity and MIS-C. While supplementation of COVID-19 cases with vitamin D showed favorable results, the effect on the outcome of the disease remains uncertain.

## Introduction and background

Coronavirus disease 2019 (COVID-19) has been typically a mild disease in children [1]. However, serious illness in children has been reported and a few of them may develop severe complications, especially without early diagnosis and treatment of the multisystem inflammatory syndrome in children (MIS-C), the pediatric hyperinflammation disorder related to the severe acute respiratory syndrome coronavirus-2 (SARS-CoV-2) [2].

Vitamin D is a fat-soluble steroid hormone with a wide range of immunoregulatory effects [3]. Recent literature data support its role as a potent regulator of innate and acquired immunity responses, its strong effect on the gene expression of antimicrobial peptides, and the inflammatory cascade [3]. The biological actions of vitamin D are executed through the vitamin D receptor (VDR), a transcription factor expressed by the cells of 30 or more different target tissues, making vitamin D a versatile hormone worth studying in different diseases [3]. Genetic changes in genes involved in the metabolism, transport, or binding of vitamin D, such as the genes encoding the VDR receptor, may result in its deficiency [4]. In fact, single nucleotide polymorphisms (SNPs) of the gene encoding VDR in children have been associated with vulnerability to acute, mainly viral, infections of the lower respiratory system, which supports the possible role of vitamin D in the immune response to viral respiratory infections [4].

The functional consequences of the genetic variants within the vitamin D pathway are reported to have a substantial impact on vitamin D levels and, thus, may confer susceptibility to infections. Still, their effect on the susceptibility of children to COVID‐19 remains unsettled. In the case of COVID-19 pathogenesis, multiple inflammatory pathways are involved, including unregulated inflammatory responses and cytokine storm [5]. Therefore, 25(OH)D status and the related genetic background may be associated with the occurrence of COVID-19 infection and adverse outcomes in children and may constitute a significant biomarker for predicting disease complications, including MIS-C. However, much remains to be elucidated regarding the association of vitamin D polymorphisms with COVID-19 susceptibility and clinical course and the impact of vitamin D supplementation on the disease in children. While the association of vitamin D deficiency with the susceptibility to COVID‐19 disease in children and adolescents is under investigation to date, it has been linked to clinical severity and higher inflammation markers in the disease in children [5,6].

Addressing the link between vitamin D genetic variants and the susceptibility of individuals to COVID-19, studies in adults have shown that *VDR* gene polymorphisms might play a critical role in the vulnerability to infection and severity of the disease [7]. In this systematic review, we aim to determine the prevalence and the effect of vitamin D deficiency in COVID-19 in children and the association between genetic variances in the vitamin D pathway and the disease severity and clinical course. In this review, we also summarize the existing data in the literature regarding the effect of vitamin D levels on the severity of MIS-C and the effect of vitamin D supplementation on the clinical outcome of COVID-19 disease.

## Review

Materials and methods

Search Strategy and Selection Criteria

This systematic review was conducted according to the recommendations of the Preferred Reporting Items for Systematic Review and Meta-Analysis (PRISMA) guidelines [8]. The studies were selected after performing a comprehensive literature search in the PubMed database without language or date restrictions. Various search strategies were piloted aiming to maximum sensitivity. The following algorithm was used: (Vitamin D OR Vit D) AND (children OR infant OR neonate OR adolescent OR pediatric) AND (COVID-19 OR SARS-CoV-2 OR COVID-2019 OR 2019-nCOV OR 2019 novel coronavirus infection OR coronavirus disease-19 OR coronavirus disease 2019 OR novel coronavirus OR mis-c OR multisystem inflammatory syndrome in children). The last search was updated on February 18, 2024.

The study selection included the following criteria: original research studies published in English and peer-reviewed journals reporting on vitamin D status or polymorphisms in vitamin D-related genes and COVID-19 infection in pediatric cases. Studies regarding the effect of vitamin D levels on the severity and prognosis of MIS-C in addition to the role of supplementation as a complementary treatment of COVID-19 disease were included. Prospective or retrospective cohort studies and cross-sectional studies were included if relevant. Exclusion criteria were non-English language publications, experimental or animal studies, review articles and case reports, studies including only adult cases, and other studies of general content.

The title and abstract of the articles were screened for relevance and duplicate records were excluded. Cited references were also reviewed to identify additional relevant studies. The first-stage screening of titles and abstracts based on our research question and the full-text articles were independently assessed by two authors according to study selection criteria. In cases of unresolved disagreement during the screening process, a third author was consulted to reach a consensus. The included studies were then reviewed in detail for data extraction.

Data Extraction and Analysis

Extraction of variables was performed using a standardized Excel form (Microsoft Corporation, Redmond, Washington, United States). The information recorded included authors, study design, population and sample size, study period, date of publication, country of the study, demographics (gender, median age), mean serum vitamin D, prevalence of low 25-hydroxyvitamin D levels, genotype analysis and association with clinical severity/risk of infection or other markers, clinical course of COVID-19 disease or MIS-C and mortality. Clinical interventions and outcomes if available were also extracted. Results were analyzed as frequencies (%) and p-value <0.05 was considered statistically significant. The heterogeneity of the included studies was reported as high and it wa assessed using Cochran’s Q test (p <0.10) and the I^2^ statistic (I^2^ >75%).

Results

Study Selection

**Figure 1:**
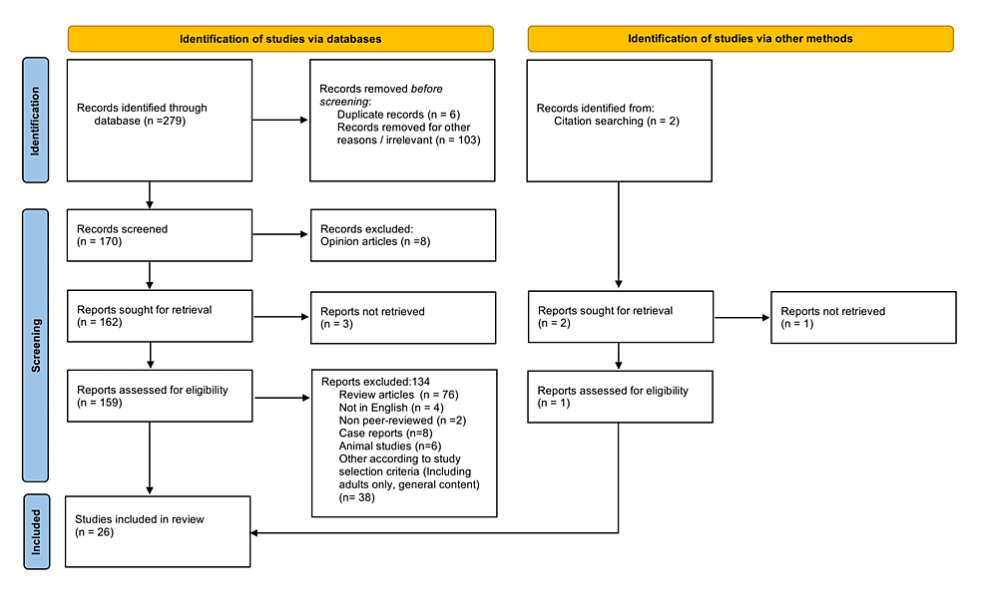
PRISMA flow diagram of selected studies. PRISMA: Preferred Reporting Items for Systematic Review and Meta-Analysis

A PRISMA flowchart (Figure 1) summarizes the literature search, study review screening, and selection. The literature search yielded a total of 279 studies, and one article was retained from citation searching. Of these, 26 studies were finally reviewed for data extraction after the removal of irrelevant studies, review articles, duplicate records, or studies that did not meet the inclusion criteria.

Characteristic Details of the Included Studies

The current systematic review provides evidence from 26 different studies published between September 2020 and May 2023. The included studies were conducted mostly in Europe (12/26), followed by Asia (10/26), America (3/26), and Africa (1/26). The total sample size varied from 15 to 987849 patients. A total of 1965 pediatric COVID-19 cases were calculated from the provided data from 22 studies and 121 MIS-C cases as provided by four studies. One of the reviewed studies included both COVID-19 and MIS-C cases [9].

Based on the information provided from 18 of the 26 included studies and 1726 pediatric COVID-19 patients, 51.76% were males and the median age of patients was 10 years (range 0.3-18.9).

Selected studies included data from pediatric patients (aged <19 years), while five studies included both pediatric and adult cases [10-14]. The selected studies included retrospective studies (16/26 with six cohort studies included), prospective studies (4/26 with two cohort studies included), cross-sectional studies (3/26), a single-center observational and a population‐based study. Additionally, a randomized controlled clinical trial was reviewed [15].

Findings of the Included Studies

Vitamin D status in pediatric COVID-19 patients: Findings regarding vitamin D status in pediatric patients with COVID-19 were reported by 23 of the 26 included studies and the proportion of patients with vitamin D deficiency was noted. Twelve of the 23 studies that reported on vitamin D status in children were conducted during the first wave of the COVID-19 pandemic, mostly between March and May 2020. Out of them, nine studies retrospectively assessed 25(OH)D levels, measured in COVID-19-infected children at admission. The remaining 11 studies were conducted during the first and/or the second COVID-19 wave. In the current review, no studies were included reporting directly on the impact of the COVID-19 lockdowns on the prevalence of vitamin D deficiency in children.

Several studies classified patients according to their serum 25(OH)D level of sufficiency, and variations were noted between the values defined as sufficient, insufficient, and deficient. Studies included in our systematic review determine vitamin D deficiency as levels of serum 25(OH)D less than 20 ng/mL (<50 nmol/L) [16-18], less than 12 ng/mL (<30 nmol/L) [9], or between 5 and 15 ng/mL [19], mild deficiency as of 21-30 ng/mL [20], and mild to moderate deficiency as of 10-24 ng/mL [21]. Two studies defined severe vitamin D deficiency as 25(OH)D levels below 10 ng/ml [21,22], and one study as less than 5 ng/mL [19]. Those with 25(OH)D levels of 21-29 ng/mL (52.5 and 72.5 nmol/l) [16,17] or 12-20 ng/mL (30-50 nmol/L) [9,23] were defined as vitamin D insufficient, and those with levels above 30 ng/ml (75 nmol/L) were considered to have normal levels [16,17].

Based on information provided by 11 studies in the present systematic review, we evaluated the proportion of COVID-19 pediatric patients with vitamin D deficiency or insufficiency. Due to wide variations in defining sufficient, insufficient, and deficient levels according to each author, a considerable value of 43.05% (n=375) was calculated in the present review as the pooled prevalence of low vitamin D levels among the included COVID-19 subjects (n=871). The pooled mean serum 25(OH)D in pediatric COVID-19 patients was evaluated as 17 ng/mL, among the included COVID-19 subjects (n=1478) from the values provided by 16 studies.

COVID-19 disease was classified as asymptomatic, mild, moderate, severe, and critical according to clinical presentation. Not all studies included all the possible patient groups. Different published classifications or national protocols were used in the included studies in order to assess clinical presentation; although quite similar, there was not a unique scoring system for all studies in the current review.

Regarding clinical presentation, six studies provided data on the proportion of patients with mild disease and were calculated to constitute 48.8% of their included cases [11,20,23-25]. Moderate and moderate-to-severe disease was addressed in seven studies and was calculated as 26.27% of the included patients [5,6,11,17,23,24]. Severe cases constituted 12% of the provided data from four studies [11,20,23,24]. Our analysis of the associated vitamin D levels with clinical severity from data provided by two studies [5,23], showed that 65.36% of the included patients with severe and moderate-to-severe disease had vitamin D insufficiency or deficiency as opposed to the 8.6% who had normal values.

In three of the 26 systematically appraised studies regarding the influence of patients’ characteristics on vitamin D status, patients' age was correlated negatively with serum 25(OH)D levels [17,26,27]. One study found no statistically significant association between age and vitamin D levels [20].

Characteristics and key data of included studies regarding the vitamin D status of COVID-19 pediatric patients and its correlation with the susceptibility to infection and clinical outcome are summarized in Table 1.

**Table 1:** Characteristic details and key findings of selected studies. PIMS-TS: pediatric inflammatory multisystem syndrome temporally associated with SARS-CoV-2; BAME: Black, Asian, and minority ethnic; MIS-C: multisystem inflammatory syndrome in children

Author, Year	Region	Population (age)	Characteristic findings
Bayramoğlu et al., 2021 [5]	Germany	Children (1-18 years)	Vitamin D deficiency is associated with clinical severity of pediatric COVID-19 and inflammation markers.
Yılmaz and Şen, 2020 [6]	Turkey	Children (1 month-18 years)	Pediatric COVID-19 cases had lower 25(OH)D levels compared to the controls. Vitamin D levels were negatively associated with fever symptoms and may be associated with COVID-19 disease by modulating inflammation and cytokine release to the virus.
Keleş et al., 2023 [9]	Turkey	Children (1 month-18 years)	Children with COVID-19 and MIS-C had insufficient vitamin D levels. Serum 25(OH)D levels may be associated with the severity of MIS-C.
D’Alessandro et al., 2023 [10]	Italy	Children and adults	Vitamin D levels were inadequate in all COVID-19 patients. Hypovitaminosis upon admission may increase the risk of COVID-19 infection and suggest a modifiable risk factor for unfavorable clinical outcomes in hospitalized patients.
Capraru et al., 2023 [11]	Romania	Children (<18 years), adults and elders	Patients’ levels of 25(OH)D were inversely related to the presence and severity of clinical symptoms, as well as ICU admission and death, demonstrating that 25(OH)D is an important biomarker for COVID-19 pediatric patients.
Basińska-Lewandowska et al., 2023 [12]	Poland	Children and adults (6-50 years)	Patients with severe 25(OH)D deficiency were more likely to develop subsequent COVID-19 infection
Kotur et al., 2021 [13]	Serbia	Children and adults	No significant correlations were observed between the associated with lower 25(OH)D levels genetic variants and the disease severity or risk for symptomatic disease in pediatric patients
Merzon et al., 2020 [14]	Israel	Children and adults (2 months-103 years)	While low plasma 25(OH)D levels were found to be an independent risk factor for COVID‐19 infection, the likelihood for hospitalization due to COVID‐19 was significant only in patients aged over 50 years.
Zurita-Cruz et al., 2022 [15]	Mexico	Children 1 month-17 years)	Vitamin D supplementation in pediatric patients appears to decrease the risk of COVID-19 progression and death.
Zeidan et al., 2023 [16]	Egypt	Children <19 years	Vitamin D deficiency and VDR Fok I FF genotype were associated with increased susceptibility to COVID-19 in children and adolescents.
Alpcan et al., 2022 [17]	Turkey	Children (1 month-17 years)	Vitamin D level was correlated negatively with age in the COVID-19 group. Vitamin D deficiency was observed in significantly more COVID-19 patients compared to the control group, and was correlated to the disease severity.
Petrovic et al., 2023 [18]	Croatia	Children (1-16 years)	MIS-C severity was significantly correlated to 25(OH)D levels. No association between 25(OH)D levels and laboratory inflammatory and cardiac markers. Vitamin D supplementation should be considered during the early stages of management of MIS-C.
Bayrak et al., 2023 [19]	Turkey	Children (1 month-18 years)	Children with COVID-19 had a significantly lower 25(OH)D vitamin level compared to the controls. No significant difference was found between COVID-19 patients with normal and low 25(OH)D levels when compared in terms of clinical severity (asymptomatic, mild, and moderate clinical course) (p= 0.418)
Karimian et al., 2022 [20]	Iran	Children (1 month-13 years)	A significant relationship was observed between vitamin D levels and levels of involvement, clinical signs and gastrointestinal complications. Clinical severity and rate of involvement had an important association with high vitamin D levels, suggesting that moderate levels of vitamin D could possibly have a better impact on the immune system.
Doğan et al., 2022 [21]	Turkey	Children (1-18 years)	COVID-19 patients had statistically significantly lower mean serum vitamin D levels than healthy children. Vitamin D supplementation should be considered as a supportive therapy in COVID-19 infection.
Torpoco Rivera et al., 2022 [22]	United States, Michigan	Children (<18 years)	Severe vitamin D deficiency was significantly correlated to severe MIS-C, including increased risk of cardiac involvement, prolonged ICU and hospital length of stay.
Heidari et al., 2022 [23]	Iran	Children (1-18 years)	A significantly positive association was observed between serum concentrations of vitamin D and levels of serum lymphocytes and neutrophils but a negative correlation with CRP, fibrinogen, and d‐dimer values. Patients with moderate or severe COVID‐19 disease had significantly lower serum vitamin D levels compared to patients with mild clinical course.
Bagiu et al., 2022 [24]	Romania	Children (0-17 years)	Vitamin D levels were indirectly moderately correlated with CRP and LDH levels, with clinical presentation and severity.
Olivé-Cirera et al., 2021 [25]	Spain	Children (8-16 years)	Risk for developing COVID-19 infection, which is low in children with neuroimmunologic disorders and not influenced by immunosuppressive therapies, was associated with low vitamin D levels.
Ozden et al., 2022 [26]	Turkey	Children (1 month-18 years)	A positive association between serum 25(OH)D levels and lymphocyte counts was observed in children with COVID-19 infection, who appeared to have a mild clinical course, suggesting the possible role of vitamin D in immune response.
Peng et al., 2022 [27]	China	Children (<18 years)	Serum 25(OH)D levels showed a significant negative association with patients' age. Vitamin D insufficiency might result in poorer clinical outcomes in children with Omicron subvariant BA.2 infection.
Katz et al., 2020 [28]	United States, Florida	Children (<18 years) and adults	An increased likelihood of COVID-19 infection was observed in patients with vitamin D deficiency.
Darren et al., 2022 [29]	United Kingdom	Children (1 month-19 years)	A predominance of PIMS-TS was found in children of BAME background, who were also found more susceptible to vitamin D deficiency.
Söbü et al., 2021 [30]	Turkey	Children (1-18 years)	Children with COVID-19 had significantly lower vitamin D levels than controls. Clinical improvement of patients with low vitamin D levels cannot be attributed to vitamin D supplementation because they also received supportive and medical treatment.
Isoldi et al., 2021 [31]	Italy	Children (<18 years)	Low vitamin D levels were observed in most of the infected patients in the study.
Karakaya Molla et al., 2021 [32]	Turkey	Children (1 month-18 years)	Vitamin D deficiency was the most common nutrient deficiency in COVID-19 patients, suggesting its possible role in increased susceptibility of children to the disease.

Vitamin D levels and COVID-19 risk and severity: The current review addressed the effect of low vitamin D levels on COVID-19 infection risk. Patients with vitamin D deficiency were more likely to develop COVID-19 infection and according to the provided data, low vitamin D levels were found to constitute an independent risk factor for the infection (p < 0.001) [12,14,28].

A total of 16 studies provided data on the correlation between serum 25(OH)D levels and COVID-19 severity in terms of the severity of clinical symptoms, hospital length of stay, and mortality in hospitalized patients aged one month to 18 years [5,6,9-11,13-17,19-21,24,27,29]. One population-based study, conducted during the first wave of the COVID-19 pandemic, assessed the likelihood of hospitalization in children over two months of age and adults [14].

A statistically significant relationship was observed between vitamin D values and the extent of pulmonary involvement, tachycardia, tachypnea, clinical signs, gastrointestinal complications, and saturation of peripheral oxygen (SpO2) levels [20]. Patients’ serum levels of 25(OH)D were inversely related to the presence and severity of clinical symptoms and the existence of respiratory manifestations including pneumonia, rhinopharyngitis, and laryngitis [6,11,24]. According to Karimian et al., clinical severity and rate of involvement had an important direct association with high vitamin D levels, suggesting that moderate levels of vitamin D could possibly have a better impact on the immune response [20]. A retrospective study reported that fever and vitamin D levels were negatively correlated among patients [6]. In a similar study, however, fever and cough were more frequent in patients with normal vitamin D levels as opposed to the vitamin D-deficient group, considering the possible role of vitamin D in the inflammatory process [17].

Vitamin D deficiency on admission was found as an independent predictor of severe COVID-19 [5]. Another study noted that low vitamin D levels on admission may constitute a modifiable risk factor for severity and mortality in hospitalized patients [10].

Unfavorable clinical outcomes were also associated with low vitamin D levels in Omicron subvariant BA.2 infection in children [27]. A weak negative correlation was found in a study regarding the severity of COVID-19 and serum 25(OH)D and the duration of hospital stay [9]. The latter was also negatively correlated with low serum 25(OH)D levels of COVID-19 patients in the study by Alpcan et al. [17]. A population‐based study including pediatric and adult cases, found that the likelihood of hospitalization due to COVID‐19 was significant only in patients aged over 50 years [14]. Another study found no difference in COVID‐19-diagnosed children and adolescents regarding vitamin D levels and disease clinical course and severity [16]. Bayrak et al. found no significant difference between COVID-19 patients with normal and low 25(OH)D levels when compared in terms of clinical severity (asymptomatic, mild, and moderate clinical course) [19].

Nine studies examined the association of vitamin D levels with inflammatory and other laboratory biomarkers in COVID-19 [5,9,16,17,19,20,23,24,26]. Two studies noted a negative correlation between serum concentrations of vitamin D with C-reactive protein (CRP) and fibrinogen levels [5,23]. Bagiu et al. stated an indirect significant association of CRP and lactate dehydrogenase (LDH) values with COVID-19 severity in patients with low values of vitamin D [24]. However, three studies found no statistically significant association of laboratory markers including ferritin and CRP with vitamin D levels [16,17,20], although CRP was found higher in the group of patients with low vitamin D levels in one of the aforementioned studies [17]. Mean lymphocyte count was found significantly lower in the same group [17], and was positively correlated with vitamin D levels in three studies [5,9,26]. Data from 21 studies including 1840 pediatric COVID-19 patients revealed a mortality rate of 0.43%. Only 2/21 studies reported deaths among hospitalized cases and cases with intensive care unit (ICU) admission, respectively [11,15].

Vitamin D levels and MIS-C severity: Next, we sought to evaluate the effect of serum vitamin D levels on the severity of MIS-C in children, indicated by clinical, laboratory, and cardiac biomarkers, as well as the length of hospitalization. Torpoco Rivera et al. [22] observed that 90% of patients with vitamin D deficiency had a severe course of the disease and Petrovic et al. [18] reported a correlation of 25(OH)D levels with MIS-C severity, including cardiovascular involvement, prolonged ICU, and hospital length of stay, although their multivariate analysis showed no independent associations. A moderate negative association with more severe MIS-C was suggested by Keleş et al. [9]. Darren et al., who also found lower mean 25(OH)D values in pediatric intensive care unit (PICU) patients as opposed to the non-PICU group, noted that the association with MIS-C severity was not statistically significant [29].

Vitamin D polymorphisms and COVID-19 risk and severity: Two studies investigated the impact of vitamin D genetic variants on susceptibility, clinical outcome, and disease severity of COVID-19 [13,16]. Zeidan et al., who investigated the VDR FokI polymorphism solely in pediatric patients, found that VDR FokI polymorphism and vitamin D deficiency could represent independent risk factors for the infection [16]. The polymorphism genotype distribution was not associated with clinical severity of COVID-19 [16]. Kotur et al. determined the significant effect of the associated with lower vitamin D levels variants DHCR7/NADSYN rs12785878, GC rs2282679, VDR rs2228570 (FokI) and CYP2R1 rs10741657 variants with severe COVID-19 in adults but no statistically significant correlations were observed between the analyzed genetic variants and the disease severity or risk for symptomatic disease in pediatric patients [13].

Vitamin D supplementation in severe COVID-19: The effect of vitamin D supplementation on COVID-19 severity was explored in a randomized controlled clinical trial appraising the effectiveness and outcomes of vitamin D supplementation in pediatric COVID-19 patients with moderate disease, as defined by requiring hospitalization or oxygen supplementation [15]. Patients were divided into the vitamin D group, which received doses of 1,000 or 2,000 IU/day according to age, and the control group without supplementation. The authors concluded that vitamin D supplementation in pediatric patients appears to decrease the risk of COVID-19 progression and death. In the study by Söbü et al., supplementation of vitamin D was administered to children with deficiency or insufficiency, and all children included in the study were cured [30]. However, it remains uncertain whether the favorable results could be attributed to vitamin D supplementation because these children were also treated with supportive and medical therapy.

Discussion

Vitamin D deficiency has been suggested to be associated with an increased risk of respiratory viral infections in children [33]. In the current review, we systematically appraised the available literature to determine the effect of vitamin D deficiency on the severity and mortality of COVID-19 infection and the reported genetic variants known to modulate susceptibility to severe COVID-19 disease. In addition, we evaluated the relationship between serum 25(OH)D levels and multisystem inflammatory syndrome related to COVID-19 in children.

Altogether, mean serum vitamin D levels were observed to be significantly low in COVID-19 cases. In this review, almost half of pediatric patients with COVID-19 presented vitamin D deficiency. This rate is in accordance with a previous report that noted a considerable number of pediatric COVID-19 patients suffering from vitamin D deficiency, with their reported prevalence also approaching the rate of 50% [34].

Vitamin D deficiency determined by serum 25(OH)D levels less than 20 ng/mL, is reported in about one-third of children worldwide, notably in adolescents [19]. Accordingly, in published data reports on the levels of vitamin D in healthy children aged 0-18 years, deficiency is found in almost 40% of them [30]. In the current review, data provided from case-control studies showed that vitamin D deficiency in healthy controls ranges from 15-17.5% [16,17] to 19.3% for severe deficiency, 56.8% for mild-moderate, and 23.8% for slight deficiency [21].

The negative association between age and serum 25(OH)D values, which was evaluated in three studies, was also addressed in a previous report in which older children including adolescents, had lower 25(OH)D serum levels [35].

In the present review, vitamin D deficiency was suggested as a risk factor for COVID-19, based on the available evidence. This finding is in accordance with another study which noted that low vitamin D levels were associated with prominent infection risk and poor prognosis in children [34]. Vitamin D insufficiency and deficiency were indicated in a significant number of cases with severe and moderate-to-severe COVID-19 disease, in this review. Although not all studies coincide on whether the presence of clinical symptoms, the likelihood of hospitalization, and the disease clinical course are significantly correlated with vitamin D deficiency, the presented evidence led us to conclude that low vitamin D levels have an impact on the risk of severe infection. Thus, this report further strengthens previously reported evidence [34].

Vitamin D is a known important modulator of innate and acquired immunity and has been shown to balance the inflammatory and macrophage responses, such as attenuating the cytokine storm, which is common in COVID-19 [3,4,7,27].

Importantly, based on the results of this review, serum 25(OH)D concentration in COVID-19 pediatric patients may affect the inflammatory and hematological parameters or vice versa, due to its role in the cytokine storm and the unregulated inflammation, and it may play a role in severe COVID-19 and lead to poor clinical outcome [7,27,36]. Although not statistically significant in all cases, more than half of the addressed studies correlated vitamin D levels with laboratory markers in pediatric COVID-19 cases.

Based on these findings, the hypothesis that the use of vitamin D as a modifiable intervention could alter COVID-19 outcomes through its protective immuno-regulatory effects emerges as crucial [37]. Indeed, it has been suggested that vitamin D supplementation may reduce clinical severity and mortality in adult patients with COVID-19 [38]. Although a beneficial effect of vitamin D supplementation in children was indicated and the provided evidence suggested a possible relationship between COVID-19 severity and vitamin D deficiency, it was not possible through the review of the available studies to establish a favorable effect of vitamin D supplementation on COVID-19 progression.

Despite the low prevalence of severe or critical COVID-19 disease in children, serious illness has been reported and a few of them may develop severe complications, especially without early diagnosis and treatment of MIS-C. In our study, although not all cases reached statistical significance, a link between MIS-C progression and severity and vitamin D deficiency was made evident. Still, it remains unclear whether vitamin D deficiency predisposes patients to severe MIS-C or constitutes a result of the disease, due to the involvement of inflammatory effects in the disease process which may result in reduced 25(OH)D [37].

Genetic changes in genes involved in the metabolism, transport, or binding of vitamin D, may result in its deficiency [4,39-44]. The major single nucleotide polymorphisms (SNPs) of the *VDR* gene have been suggested to be associated with an increased risk of lower respiratory tract and viral infections in children [44-48]. In parallel, published evidence supports a major role of the rs10741657 polymorphism of the *CYP2R1* gene in vitamin D deficiency [42,43,49,50].

To our knowledge, only two studies have addressed the effect of vitamin D genetic variants on COVID-19 risk of infection and severity in children [13,16].

Based on the evidence provided by two studies [13,16], an evident relationship between the VDR FokI polymorphism and COVID-19 severity or clinical course could not be established, but it may constitute an independent risk factor for susceptibility to COVID-19 [16]. Other genetic variants related to the status and bioavailability of vitamin D including the DHCR7/NADSYN rs12785878, GC rs2282679, and CYP2R1 rs10741657 could not be significantly related to the symptomatic or severe COVID-19 in children [13]. However, since recognition of the genetic susceptibility of children to infection could contribute to early treatment through personalized strategies but still remains relatively unexplored in the literature, more primary studies are required to explore and substantiate this hypothesis.

Although this review provides a broad systematic literature search, assessment of study selection by two independent authors, and review in detail for data extraction, it has several limitations, including the heterogeneity of the included studies in terms of study design, participant characteristics, and mainly the wide variation between the 25(OH)D levels determined as sufficient, insufficient, and deficient by each author. Thus, this difference can lead to a measurement bias regarding the calculated pooled prevalence of vitamin D deficiency and the pooled mean value of the participants, which considerably limited the analysis and interpretation of the provided evidence. Furthermore, due to limited published data and the unavailability of randomized controlled trials, we could not explore the impact of vitamin D supplementation in pediatric COVID-19 patients.

## Conclusions

Our findings demonstrated that most COVID-19 patients had suboptimal or low vitamin D serum levels. The estimate of suboptimal and low vitamin D levels in COVID-19 in children was provided in this systematic review, as well as the available evidence regarding its association with the clinical course of the disease and complications. While it appears that the infection has a mild course in children, 25(OH)D levels may suggest an independent risk factor for susceptibility to infection and adverse outcomes in the pediatric population. In addition, vitamin D, probably acting as an important immunomodulatory agent, may constitute a significant biomarker in MIS-C associated with the disease severity. While there is recent evidence that vitamin D genetic variants are associated with COVID-19 severity in adults, findings provided in the review underline the need for further investigation of vitamin D genetic polymorphisms distribution and their possible role as disease risk factors and markers of severe clinical course. The effect of vitamin D supplementation on COVID-19 in children needs to be further explored in future clinical trials.

## References

[1] Ludvigsson JF (2020). Systematic review of COVID-19 in children shows milder cases and a better prognosis than adults. Acta Paediatr.

[2] Kabeerdoss J, Pilania RK, Karkhele R, Kumar TS, Danda D, Singh S (2021). Severe COVID-19, multisystem inflammatory syndrome in children, and Kawasaki disease: immunological mechanisms, clinical manifestations and management. Rheumatol Int.

[3] Gombart AF (2009). The vitamin D-antimicrobial peptide pathway and its role in protection against infection. Future Microbiol.

[4] Bahrami A, Sadeghnia HR, Tabatabaeizadeh SA (2018). Genetic and epigenetic factors influencing vitamin D status. J Cell Physiol.

[5] Bayramoğlu E, Akkoç G, Ağbaş A, Akgün Ö, Yurdakul K, Selçuk Duru HN, Elevli M (2021). The association between vitamin D levels and the clinical severity and inflammation markers in pediatric COVID-19 patients: single-center experience from a pandemic hospital. Eur J Pediatr.

[6] Yılmaz K, Şen V (2020). Is vitamin D deficiency a risk factor for COVID-19 in children?. Pediatr Pulmonol.

[7] Abdollahzadeh R, Shushizadeh MH, Barazandehrokh M (2021). Association of Vitamin D receptor gene polymorphisms and clinical/severe outcomes of COVID-19 patients. Infect Genet Evol.

[8] Page MJ, McKenzie JE, Bossuyt PM (2021). The PRISMA 2020 statement: an updated guideline for reporting systematic reviews. BMJ.

[9] Ekemen Keleş Y, Yılmaz D, Taşar S (2023). Can serum 25-hydroxy vitamin d levels predict the severity of multisystem inflammatory syndrome in children and COVID-19?. J Clin Res Pediatr Endocrinol.

[10] D'Alessandro A, Ciavardelli D, Pastore A (2023). Contribution of vitamin D(3) and thiols status to the outcome of COVID-19 disease in Italian pediatric and adult patients. Sci Rep.

[11] Capraru ID, Vulcanescu DD, Bagiu IC (2023). COVID-19 biomarkers comparison: children, adults and elders. Medicina (Kaunas).

[12] Basińska-Lewandowska M, Lewandowski K, Horzelski W, Lewiński A, Skowrońska-Jóźwiak E (2023). Frequency of COVID-19 infection as a function of vitamin D levels. Nutrients.

[13] Kotur N, Skakic A, Klaassen K (2021). Association of vitamin D, zinc and selenium related genetic variants with COVID-19 disease severity. Front Nutr.

[14] Merzon E, Tworowski D, Gorohovski A, Vinker S, Golan Cohen A, Green I, Frenkel-Morgenstern M (2020). Low plasma 25(OH) vitamin D level is associated with increased risk of COVID-19 infection: an Israeli population-based study. FEBS J.

[15] Zurita-Cruz J, Fonseca-Tenorio J, Villasís-Keever M, López-Alarcón M, Parra-Ortega I, López-Martínez B, Miranda-Novales G (2022). Efficacy and safety of vitamin D supplementation in hospitalized COVID-19 pediatric patients: a randomized controlled trial. Front Pediatr.

[16] Zeidan NM, Lateef HM, Selim DM (2023). Vitamin D deficiency and vitamin D receptor FokI polymorphism as risk factors for COVID-19. Pediatr Res.

[17] Alpcan A, Tursun S, Kandur Y (2021). Vitamin D levels in children with COVID-19: a report from Turkey. Epidemiol Infect.

[18] Petrovic D, Benzon B, Srsen S, Polic B, Vukovic Novogradec A, Milic P, Markic J (2023). The impact of vitamin D levels on clinical manifestations of multisystem inflammatory syndrome in children: a cross-sectional study. Life (Basel).

[19] Bayrak H, Öztürk D, Bolat A, Ünay B (2023). Association between vitamin D levels and COVID-19 infection in children: a case-control study. Turk Arch Pediatr.

[20] Karimian P, Tahami MS, Sayyahfar S, Aghajani Delavar M (2022). Association of vitamin D and severity of COVID-19 in children. Eur J Transl Myol.

[21] Doğan A, Dumanoğlu Doğan İ, Uyanık M, Köle MT, Pişmişoğlu K (2022). The clinical significance of vitamin D and zinc levels with respect to immune response in COVID-19 positive children. J Trop Pediatr.

[22] Torpoco Rivera D, Misra A, Sanil Y, Sabzghabaei N, Safa R, Garcia RU (2022). Vitamin D and morbidity in children with multisystem inflammatory syndrome related to Covid-19. Prog Pediatr Cardiol.

[23] Heidari S, Mohammadi S, Fathi M, Cigary S, Alisamir M, Mirkarimi M, Aminzadeh M (2022). Association of vitamin D status with COVID-19 disease severity in pediatric patients: a retrospective observational study. Health Sci Rep.

[24] Bagiu IC, Scurtu IL, Horhat DI (2022). COVID-19 inflammatory markers and vitamin D relationship in pediatric patients. Life (Basel).

[25] Olivé-Cirera G, Fonseca E, Cantarín-Extremera V (2022). Impact of COVID-19 in immunosuppressed children with neuroimmunologic disorders. Neurol Neuroimmunol Neuroinflamm.

[26] Ozden A, Doneray H, Hafize Erdeniz EH, Altinkaynak K, Igan H (2022). Clinical and laboratory findings by serum vitamin D levels in children with COVID-19. Eurasian J Med.

[27] Peng D, Huang H, Liu Z, Gao Y, Liu Y (2022). Vitamin D levels and clinical outcomes of SARS-CoV-2 Omicron subvariant BA.2 in children: a longitudinal cohort study. Front Nutr.

[28] Katz J, Yue S, Xue W (2021). Increased risk for COVID-19 in patients with vitamin D deficiency. Nutrition.

[29] Darren A, Osman M, Masilamani K (2022). Vitamin D status of children with paediatric inflammatory multisystem syndrome temporally associated with severe acute respiratory syndrome coronavirus 2 (PIMS-TS). Br J Nutr.

[30] Söbü E, Karaaslan A, Çetin C (2021). Vitamin D levels of COVID-19 positive sypmtomatic pediatric cases. J Curr Pediatr.

[31] Isoldi S, Mallardo S, Marcellino A (2021). The comprehensive clinic, laboratory, and instrumental evaluation of children with COVID-19: a 6-months prospective study. J Med Virol.

[32] Karakaya Molla G, Ünal Uzun Ö, Koç N, Özen Yeşil B, Bayhan Gİ (2021). Evaluation of nutritional status in pediatric patients diagnosed with Covid-19 infection. Clin Nutr ESPEN.

[33] Raju A, Luthra G, Shahbaz M (2022). Role of vitamin D deficiency in increased susceptibility to respiratory infections among children: a systematic review. Cureus.

[34] Shah K, Varna VP, Pandya A, Saxena D (2021). Low vitamin D levels and prognosis in a COVID-19 pediatric population: a systematic review. QJM.

[35] Alvares MA, Ribas BH, Miranda GB, Miranda RB, Natário EM, D'Angelo IS, Rullo VE (2022). Clinical prognosis of coronavirus disease 2019 in children and vitamin D levels: a systematic review. Rev Assoc Med Bras (1992).

[36] Daneshkhah A, Agrawal V, Eshein A, Subramanian H, Roy HK, Backman V (2020). Evidence for possible association of vitamin D status with cytokine storm and unregulated inflammation in COVID-19 patients. Aging Clin Exp Res.

[37] Feketea G, Vlacha V, Bocsan IC, Vassilopoulou E, Stanciu LA, Zdrenghea M (2021). Vitamin D in corona virus disease 2019 (COVID-19) related multisystem inflammatory syndrome in children (MIS-C). Front Immunol.

[38] Hosseini B, El Abd A, Ducharme FM (2022). Effects of vitamin D supplementation on COVID-19 related outcomes: a systematic review and meta-analysis. Nutrients.

[39] Martineau AR, Forouhi NG (2020). Vitamin D for COVID-19: a case to answer?. Lancet Diabetes Endocrinol.

[40] Tomei S, Singh P, Mathew R (2020). The role of polymorphisms in vitamin D-related genes in response to vitamin D supplementation. Nutrients.

[41] Bouillon R, Schuit F, Antonio L, Rastinejad F (2019). Vitamin D binding protein: a historic overview. Front Endocrinol (Lausanne).

[42] Harishankar M, Sampath P, Sriram M (2021). Association of CYP2R1 gene polymorphisms in pulmonary tuberculosis. Meta Gene.

[43] Duan L, Xue Z, Ji H, Zhang D, Wang Y (2018). Effects of CYP2R1 gene variants on vitamin D levels and status: a systematic review and meta-analysis. Gene.

[44] Zacharioudaki M, Messaritakis I, Galanakis E (2021). Vitamin D receptor, vitamin D binding protein and CYP27B1 single nucleotide polymorphisms and susceptibility to viral infections in infants. Sci Rep.

[45] Abouzeid H, Abdelaal NM, Abdou MA (2018). Association of vitamin D receptor gene FokI polymorphism and susceptibility to CAP in Egyptian children: a multicenter study. Pediatr Res.

[46] Li W, Guo L, Li H (2015). Polymorphism rs2239185 in vitamin D receptor gene is associated with severe community-acquired pneumonia of children in Chinese Han population: a case-control study. Eur J Pediatr.

[47] Roth DE, Jones AB, Prosser C, Robinson JL, Vohra S (2008). Vitamin D receptor polymorphisms and the risk of acute lower respiratory tract infection in early childhood. J Infect Dis.

[48] McNally JD, Sampson M, Matheson LA, Hutton B, Little J (2014). Vitamin D receptor (VDR) polymorphisms and severe RSV bronchiolitis: a systematic review and meta-analysis. Pediatr Pulmonol.

[49] Thacher TD, Fischer PR, Singh RJ, Roizen J, Levine MA (2015). CYP2R1 mutations impair generation of 25-hydroxyvitamin D and cause an atypical form of vitamin D deficiency. J Clin Endocrinol Metab.

[50] Cheng JB, Levine MA, Bell NH, Mangelsdorf DJ, Russell DW (2004). Genetic evidence that the human CYP2R1 enzyme is a key vitamin D 25-hydroxylase. Proc Natl Acad Sci U S A.

